# Protocol to infect differentiated human primary bronchial epithelial cells with live mycobacteria and determine intracellular load

**DOI:** 10.1016/j.xpro.2025.103871

**Published:** 2025-06-04

**Authors:** Amy M. Barclay, Kimberley V. Walburg, Dennis K. Ninaber, Tom H.M. Ottenhoff, Pieter S. Hiemstra, Anne M. van der Does, Simone A. Joosten

**Affiliations:** 1 Leiden University Center for Infectious Diseases (LUCID), Leiden University Medical Center (LUMC), Leiden 2333ZA, the Netherlands; 2 PulmoScience Lab, Department of Pulmonology, LUMC, Leiden 2333ZA, the Netherlands

**Keywords:** cell culture, flow cytometry, immunology, microbiology, microscopy

## Abstract

Lung epithelial cells present the first line of defense against pathogens like *Mycobacterium tuberculosis* and other related species. Studying their responses is instrumental to understand early infection stages of tuberculosis. We present a protocol to study the infection potential of mycobacteria in differentiated primary human bronchial epithelial cell cultures. We describe mycobacterial and epithelial cell culture techniques. We then detail how to perform infections in epithelial cells and determine intracellular bacterial load using flow cytometry and colony-forming unit assays.

For complete details on the use and execution of this protocol, please refer to Barclay et al.^1^^,^^2^

## Highlights

• Steps for culturing and differentiating human primary bronchial epithelial cells
• Assess mycobacterial infection efficiency in epithelial cells
• Determine intracellular infection with fluorescent microscopy

## Abstract (editor highlights)

Publisher’s note: Undertaking any experimental protocol requires adherence to local institutional guidelines for laboratory safety and ethics.

## Graphical abstract

**Figure:**
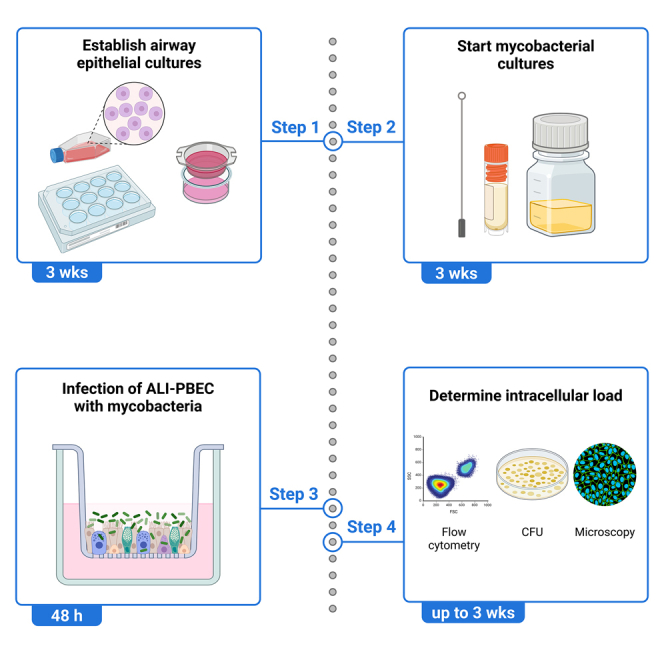

## Before you begin

Mycobacteria like *M. tuberculosis* (Mtb) and non-tuberculous mycobacteria (NTM) like *M. avium* (Mav) pose a significant threat to global health. The incidence of mycobacterial lung diseases is expected to rise, and there is currently no vaccine that offers full protection. Studying lung epithelial responses to mycobacteria is important to understand early infection events and further improve vaccine development. This protocol describes the specific steps to initiate mycobacterial cultures from cryopreserved stocks and maintaining them over time. Then, we describe steps to establish cultures of primary human bronchial epithelial cells (PBEC) and differentiate those by culture at the air-liquid interface (ALI). These differentiated ALI-PBEC cultures contain the dominant cell types of the bronchial epithelium, and ciliary activity can be observed. Next, we detail how to perform infections on ALI-PBEC using mycobacteria, and how to eliminate extracellular bacteria. Finally, we provide instructions on determining the intracellular mycobacterial load in ALI-PBEC via flow cytometry, colony forming unit (CFU) assay and fluorescence microscopy. Combining these three techniques offers detailed insight into infection efficiency of mycobacteria in airway epithelial cells and may be transferrable to other lung region cultures (e.g. nasal, alveolar). Culture media and agar plates should be prepared as described below before starting this protocol.

### Preparation of 7H9 bacterial culture medium

**Timing: 1 h**

1. Prepare the 7H9 broth.
  a. Weigh 4.7 g 7H9 broth powder stock and add to a 1 L glass bottle.
  b. Add 900 mL Milli-Q water, 2 mL glycerol and 0.5 mL Tween-80 (final concentrations 0.2% and 0.05%, respectively).
  c. Mix well by inverting the solution a few times.
  d. Sterilize the solution using an autoclave and allow to cool down to 45°C before proceeding.
2. During step 1d, prepare Middlebrook ADC supplement.
  a. Dissolve 50 g BSA, 20 g dextrose and 0.03 g catalase in 1 L Milli-Q water.
  b. Sterilize the solution using a 0.2 μm cellulose membrane syringe filter (Corning).
3. Prepare 7H9 medium.
  a. Add 100 mL ADC supplement to the 7H9 broth from step 1 (final concentration 10%).
4. 7H9 medium must be stored at 2-8°C and used within 2 months.

### Preparation of 7H10 agar

**Timing: 2 h**

5. Prepare the 7H10 agar.
  a. Weigh 19 g 7H10 agar and add to a 1 L glass bottle.
  b. Add 900 mL Milli-Q water and 5 mL glycerol (final concentration 0.5%).
  c. Mix well by inverting the solution a few times.
  d. Sterilize the solution using an autoclave and let it cool down to 45°C before proceeding.
6. Meanwhile, put the Middlebrook OADC supplement (Thermo Fisher Scientific) at 18-21°C.
7. Once the 7H10 agar has cooled, add 100 mL OADC supplement (final concentration 10%).
8. Add the 7H10 agar to sterile 10 × 10 cm square petri dishes.
  a. Pipet 35 mL 7H10 agar per petri dish. Approximately 28 petri dishes can be made using 1 L agar.
  b. Allow the 7H10 agar to set at least 12 h at 18°C–21°C.
9. Solidified 7H10 agar plates must be stored at 2°C–8°C and used within 2 months.

### Preparation of cBD medium for epithelial cell culture

**Timing: 30 min**

The cBD epithelial cell culture medium consists of equal parts Dulbecco’s Modified Eagle’s Medium (DMEM) and Bronchial Epithelial Cell Medium-basal (BEpiCM), supplemented with penicillin/streptomycin, HEPES, Glutamax, and bronchial epithelial cell growth supplement (BEpiCGS).

10. Add 5 mL penicillin/streptomycin (100×, final concentration 1×) to 500 mL of BEpiCM-b.
11. Add 5 mL penicillin/streptomycin (100×, final concentration 1×), 12.5 mL HEPES (1 M, final concentration 25 mM) and 5 mL Glutamax (100×, final concentration 1×) to 500 mL DMEM.
12. In a sterile 500 mL bottle, mix 250 mL BEpiCM-b prepared in step 10 and 250 mL DMEM prepared in step 11.
13. Add 5 mL of BEpiCGS (final concentration 1%) to the mixture prepared in step 11.
14. Aliquot 40 mL portions of the medium prepared in step 12 in centrifuge tubes. The medium can be stored for 6 months at −20°C or at 4°C for 4 weeks.

***Note:*** To prepare antibiotics-free cBD medium, skip step 10, and do not add penicillin/streptomycin to DMEM during step 11. Then continue with the protocol from step 12.

### Preparation of soft trypsin

**Timing: 10 min**

15. Prepare soft trypsin.
  a. Add 150 mg Trypsin, 50 mg EDTA and 500 mg Glucose to 500 mL PBS.
  b. Set the pH to 7.45.
  c. Sterilize the solution using a 0.2 μm filter.
  d. Add 5 mL penicillin/streptomycin (100×).
  e. Aliquot 40 mL portions in centrifuge tubes and store at −20°C up to 6 months.
16. Prepare Soybean Trypsin Inhibitor (SBTI) solution in KSFM.
  a. Dissolve 500 mg SBTI in 454.5 mL KSFM.
  b. Add 5 mL penicillin/streptomycin (100×) to this solution.
  c. Sterilize the solution using a 0.2 μm filter.
  d. Aliquot 40 mL portions in centrifuge tubes and store at −20°C.

### Institutional permissions

Approval from your local ethics committee is required to perform experiments using patient-derived tissues or cells. Bronchial epithelial cells were isolated from lung tissue that appeared normal on a macroscopic level, obtained from patients undergoing resection surgery for lung cancer at the Leiden University Medical Center (LUMC), the Netherlands. Patients from whom this lung tissue was derived were enrolled in the biobank via a no-objection system for coded anonymous further use of such tissue (www.coreon.org). Samples from this Biobank were approved for research use by the institutional medical ethical committee (BB22.006/AB/ab). Since 01-09-2022, patients are enrolled in the biobank using written informed consent in accordance with local regulations from the LUMC biobank with approval by the institutional medical ethical committee (B20.042/KB/kb). Local regulations regarding biosafety may also apply when working with pathogenic mycobacteria.

## Key resources table

**Table:** 

REAGENT or RESOURCE	SOURCE	IDENTIFIER
**Antibodies**		

Anti-SCGB1A1 antibody produced in rabbit (used 50 times diluted)	Sigma-Aldrich	HPA031828
Human EpCAM/TROP-1 antibody produced in goat (used 20 times diluted)	R&D Systems	AF960
Monoclonal anti-tubulin, acetylated antibody produced in mouse (used 200 times diluted)	Sigma-Aldrich	T6793
Lab Vision Mucin 5AC (MUC5AC)/gastric Mucin Ab-1, mouse monoclonal antibody (used 200 times diluted)	Thermo Fisher Scientific	MS-145-P1
Recombinant anti-p63 antibody (EPR5701) (used 200 times diluted)	Abcam	ab124762
Donkey anti-goat IgG (H+L) highly cross-adsorbed secondary antibody, Alexa Fluor 405 (used 200 times diluted)	Thermo Fisher Scientific	A48259
Donkey anti-mouse IgG (H+L) cross-adsorbed secondary antibody, Alexa Fluor 647 (used 200 times diluted)	Thermo Fisher Scientific	A-31571
Donkey anti-rabbit IgG (H+L) highly cross-adsorbed secondary antibody, Alexa Fluor 647 (used 200 times diluted)	Thermo Fisher Scientific	A-31573

**Biological samples**		

Primary human bronchial epithelial cells	Isolated from macroscopically normal lung tissue obtained from patients undergoing resection surgery for lung cancer at the Leiden University Medical Center (LUMC). Patients were enrolled in a biobank using written informed consent in accordance with local regulations from the LUMC biobank and with approval by the institutional medical ethical committee (B20.042/Ab/ab and B20.042/Kb/kb).	N/A

**Chemicals, peptides, and recombinant proteins**		

ProLong Diamond antifade mountant	Thermo Fisher Scientific	P36965
LIVE/DEAD fixable violet dead cell stain kit, for 405 nm excitation	Thermo Fisher Scientific	L34955
Dulbecco’s modified Eagle’s medium (DMEM) + 4,500 mg/L D-glucose	STEMCELL Technologies	36250
Bronchial epithelial cell medium-basal (BEpiCM-b)	ScienCell Research Laboratories	SCC3211-b
Bronchial epithelial cell growth supplement (BEpiCGS)	ScienCell Research Laboratories	SCC3262
HEPES (1 M)	ScienCell Research Laboratories	600212-500
GlutaMAX	Thermo Fisher Scientific	35050038
EC 23 synthetic retinoid acid receptor agonist	Tocris Bioscience	4011
Penicillin/streptomycin (100×) (10,000 U/mL/10,000 μg/mL)	Thermo Fisher Scientific	15140122
Bovine albumin fraction V (BSA, 7.5% solution)	Thermo Fisher Scientific	15260037
PureCol (3 mg/mL)	Advanced BioMatrix	5005-b
Soybean trypsin inhibitor (SBTI)	Sigma-Aldrich	T-9128
Soft trypsin	Thermo Fisher Scientific	27250-018
TrypLE express enzyme (1×), phenol red	Thermo Fisher Scientific	12605010
Air-liquid interface medium	PromoCell	c-21080
Glucose	VWR BDH Chemicals	101174Y
EDTA	VWR BDH Chemicals	443885J
Keratinocyte serum-free medium (KSFM)	Thermo Fisher Scientific	17005-059
Dextrose	Sigma-Aldrich	D9434
Catalase from bovine liver	Sigma-Aldrich	C1345-10G
BD Difco dehydrated culture media: Middlebrook 7H9 broth	Thermo Fisher Scientific	11753473
BD Difco dehydrated culture media: Middlebrook 7H10 agar	Thermo Fisher Scientific	11799042
BD BBL Middlebrook OADC enrichment	Thermo Fisher Scientific	17434923
Glycerol	Sigma-Aldrich	G9012
Tween 80	Sigma-Aldrich	P1754

**Other**		

Falcon round-bottom polystyrene test tubes with cell strainer snap cap, 5 mL	STEMCELL Technologies	38030
12-well plate 0.4 μm PET clear extended culture (FP), used for air-liquid interface culture	cellQART	9310424
Gosselin octagonal PET bottle, 125 mL, graduated, 31 mm tamper-evident cap, sterile, assembled	Corning	P125B-50
Cellulose membrane (surfactant-free with prefilter) syringe filters with 0.2 μm pore size, used to filter sterilize medium components	Corning	CLS431218

## Materials and equipment

**Table:** 7H9 bacterial culture medium

Reagent	Final concentration	Amount
Milli-Q water	N/A	900 mL
7H9 broth	0.47%	4.7 g
Glycerol	0.2%	2 mL
Tween-80	0.05%	0.5 mL
Middlebrook ADC supplement (750 mM BSA + 110 mM dextrose + 130 μM catalase in Milli-Q water)	10%	100 mL
Total	N/A	1 L

Store at RT for 2 weeks or at 4°C and use within 2 months.

**Table:** 7H10 agar

Reagent	Final concentration	Amount
Milli-Q water	N/A	900 mL
7H10 agar	1.9%	19 gr
Glycerol	0.5%	5 mL
Middlebrook OADC supplement	10%	100 mL
Total	N/A	1 L

Use approximately 35 mL of agar per petri dish. Store at 4°C and use within 2 months.

**Table:** cBD epithelial cell culture medium

Reagent	Final concentration	Amount
BEpiCM-b with supplements	45%	250 mL
Penicillin/streptomycin (100×)	1×	2.5 mL
DMEM with supplements	45%	250 mL
Penicillin/streptomycin (100×)	1×	2.5 mL
HEPES (1 M)	25 mM	6.25 mL
Glutamax (100×)	1×	2.5 mL
BEpiCGS	10%	5 mL
Total	N/A	500 mL

Store for 6 months at −20°C or at 4°C for 4 weeks.

## Step-by-step method details

### Part 1: Initiating mycobacterial cultures from cryopreserved stocks and maintaining mycobacterial cultures

**Timing: 2–3 weeks**

In this step, we describe how to start mycobacterial cultures from cryopreserved stocks and how to maintain mycobacterial cultures over time. For Initiating mycobacterial cultures from cryopreserved stocks follow steps 1–9 and for maintaining mycobacterial cultures follow steps 10–11.

***Note:*** Mycobacterial strains are kept in 7H9 medium containing 25% glycerol and stored at −70°C. As they are relatively slow-growing species, starting up a bacterial culture may take several weeks. Growth speed is mycobacterial species-dependent, and some species may take a full three weeks to grow to an appropriate density while others are faster.

***Note:*** All procedures described in the following protocols are performed in biological safety cabinets under sterile working conditions, while wearing personal protective equipment in accordance with biosafety level 3 (BSL-3) regulations. BSL-3 containment is required for virulent Mtb strains. BSL-2 containment is often sufficient for less virulent species like *M. avium* or *M. bovis* (BCG). Refer to your local regulations and permits to determine what biosafety level is appropriate for your strains.

1. Prepare a sterile 50 mL conical centrifuge tube with 1–5 mL 7H9 medium.
2. Add mycobacteria to the tube.
  a. Scrape the frozen surface of the culture in the cryotube using an inoculation needle.
  b. Swirl the inoculation needle in the 50 mL centrifuge tube.
  c. Alternatively: thaw cryotube and pipette 100 μL bacterial culture into the tube.
3. Incubate in a dry incubator without water source to maintain humidity, at 37°C for 1 week without shaking.
4. On day 7, add 9 mL 7H9 medium to the tube containing mycobacteria.
5. Transfer the culture to a shaking incubator set at 35 × g.
6. Incubate at 37°C for 1 week in a dry incubator.
7. On day 14, add 15 mL 7H9 medium to the tube of mycobacteria.
8. Transfer the culture to a Gosselin Octagonal PET bottle (Corning).
9. Incubate at 37°C for 1 week in a dry incubator.

***Note:*** Some strains may be transformed with certain plasmids that require the addition of antibiotics. These should be added in the appropriate concentration during step 8.

***Note:*** Since mycobacteria are slow-growing bacteria and starting new cultures takes several weeks, it is necessary to maintain bacterial cultures throughout the course of experiments. It is recommended to restart cultures after three months of use.

10. Measure OD_600_ of mycobacteria.
  a. Prepare 1 mL cuvettes with 800 μL 7H9 medium.
  b. Gently shake bacterial culture to homogenize, then add 200 μL bacterial culture to cuvettes.
  c. Measure OD_600_ using a spectrophotometer.
11. Dilute cultures to OD_600_ = 0.25 in 25–50 mL medium.
  a. Calculate the dilution factor for the cultures using the measured OD_600_.
  b. Prepare a plastic flask with screw cap with the appropriate amount of 7H9 medium.
  c. Transfer the calculated amount of bacterial culture to the flask.
  d. Add antibiotics in the appropriate concentration if the strain requires it.

***Note:*** Mtb cultures should be split as described in steps 10-11 once every seven days. Species with a faster growth rate may require splitting multiple times per week, as the stationary growth phase of these bacteria is reached more quickly.

**CRITICAL:** When performing infection experiments, split mycobacteria 1 day prior to the experiment to ensure bacteria are in the optimal log growth phase during infection.

### Part 2: Primary bronchial epithelial cell culture

**Timing: 3 weeks**

In this step, primary bronchial epithelial cells are cultured at air-liquid interface for 2 weeks, which results in differentiation of a cell layer that contains all major bronchial epithelial cell types, visible ciliary activity and the formation of a pseudostratified epithelial layer. Optimal differentiation time may need adjustment depending on the cell source.

***Note:*** This protocol is an optimization of a previous protocol by Ninaber et al.^3^ Please refer to that publication for detailed steps on isolation, cryopreservation and thawing of primary human bronchial epithelial cells (PBEC). Generally, PBEC are cultured 1 week in a flask, and 1 week on submerged inserts before air exposure. The protocol below describes only the establishment of air-liquid interface cultures with PBEC.

**CRITICAL:** All incubation steps that include epithelial cells should be performed at 37°C in a humidified incubator with 5% CO_2_.

12. Prepare PET clear extended culture inserts with an inner diameter of 11.9 mm and 0.4 μm pores (CellQART) for seeding cells. Inserts are provided in prefilled, specially designed 12-well cell culture plates.
  a. Prepare coating solution: add 100 μL PureCol (3 mg/mL) and 100 μL BSA (0.1%) to 10 mL cold PBS.
  b. Coat inserts by adding 400 μL coating solution per insert.
  c. Incubate 12–16 h at 37°C in a cell culture incubator.
13. Trypsinize PBEC to make a single cell suspension.
  a. Add 2 mL soft trypsin to the T75 flask.
  b. Incubate 5–10 min at 37°C.
  c. Inspect whether cells have detached from the plastic under a microscope.
14. Collect cells in SBTI to neutralize trypsin.
  a. Prepare 25 mL centrifuge tubes with 4 mL SBTI each.
  b. Directly transfer cells in soft trypsin from T75 flasks to the tubes.
  c. Rinse T75 flasks with some SBTI and add this to the tubes to collect remaining cells.
15. Centrifuge cells at 230 × g for 7 min. Remove supernatant.
16. Resuspend cells in 6 mL cBD medium.
17. Count cells using a hemocytometer or automated cell counter.

***Note:*** Include a live/dead stain like trypan blue by mixing cells 1:1 with the staining solution before counting.

18. Add 40,000 cells per insert.
  a. Dilute cell suspension to 80,000 cells/mL in cBD medium supplemented with 1 nM EC 23.
  b. Remove coating solution from inserts.
  c. Add 500 μL cell suspension per insert.
  d. Add 1.5 mL cBD medium supplemented with 1 nM EC 23 to the wells underneath the inserts.

***Optional:*** ALI-PBEC cultures can be established using a mixed donor population as well: mix equal number of cells from up to five donors to reach a total of 300,000 cells/mL (seeding number: 150.000 per insert).^4^ This high number of cells ensures that original ratios of donors in the mix are preserved. Continue protocol as described in step 18b.

19. Incubate cells at 37°C until the cultures have reached 100% confluency. Change the medium three times per week: 500 μL in the insert, 1.5 mL in the well.

***Note:*** In general, cultures reach confluency within 5–7 days. Visually inspect confluency using an inverted microscope before moving to the next steps.

20. Two days after reaching confluency, transfer cells to air-liquid interface.
  a. Remove medium from insert and wells.
  b. Add 1 mL cBD medium supplemented with 50 nM EC 23 to each well, placing the pipette tip in the gap between the insert and the wall of the well. This will submerge the basal compartment of the model so the medium touches the filter membrane.
  c. Do not add medium in the insert, leave the insert exposed to air on the apical side.

**CRITICAL:** Ensure that no air bubbles are trapped below the insert in the basal compartment. Air bubbles will prevent cells in that area from growing as they do not have access to the cBD medium.

21. Culture cells at air-liquid interface for at least 14 days.
  a. Change medium three times per week.
  b. Remove excess mucus and cell debris by adding 200 μL warm PBS to the apical side of each insert for 10 min at 37°C. Then aspirate PBS.
22. Incubate ALI-PBEC with antibiotics-free cBD supplemented with 50 nM EC 23 at least 48 h before performing infection experiments.

***Note:*** Evaluate the quality and differentiation of the PBEC cell layer using an inverted microscope. The cell layer should be confluent, without gaps. If cell differentiation was successful, ciliated cells with beating cilia can be observed. In addition, the presence of gel-like mucus is an indicator of goblet cell differentiation. Trans-epithelial electrical resistance (TEER) can also be measured for quality control of the cultures. These techniques have been described in detail by Ninaber et al.^3^

***Note:*** We have previously determined that after two weeks of differentiation, on average, our ALI-PBEC cultures contain 10^6^ cells. This can be determined by preparing single-cell suspensions (as described in Part 4, step 33–26 of this protocol) and counting cells manually using counting chambers, or with an automated cell counter.

### Part 3: Infection of ALI-PBEC with mycobacteria

**Timing: 1 h**

During this step, bronchial epithelial cells cultured in Part 2 are infected with mycobacteria cultured in Part 1 to analyses intracellular bacterial load. Be sure to incubate the cells with antibiotics-free medium at least 48 h prior to infection as described in step 22.

**CRITICAL:** All incubation steps that include epithelial cells should be performed at 37°C in a humidified incubator with 5% CO_2_.

23. Replace medium of ALI-PBEC cultures.
  a. Remove cBD medium from the basal compartment of the wells.
  b. Add 1 mL warm antibiotics-free cBD medium supplemented with 50 nM EC 23 to each well.
24. Prepare bacterial suspensions in the desired multiplicity of infection (MOI), i.e. the ratio of bacteria to epithelial cells.
  a. Dry 7H10 agar plates at 37°C in a dry incubator for at least 1 h before plating bacterial suspensions in step 25.
  b. Gently shake bacterial cultures to homogenize. For each bacterial strain, prepare three dilutions in cuvettes: undiluted, diluted two times, and diluted five times in 7H9 medium.
  c. Measure OD_600_ of the strains.
  d. Calculate the number of bacteria per mL of culture by correlating the OD_600_ to CFU/mL to obtain the OD factor.
  e. Calculate the number of bacteria needed to obtain MOI 100 (100 bacteria per epithelial cell) and how much to dilute or concentrate the bacterial culture. An example is listed in Table 1.
  f. Collect the required amount of bacterial culture in 15 mL or 50 mL centrifuge tubes and centrifuge for 10 min at 805 × g. Carefully remove supernatant.
  g. Resuspend bacteria in the appropriate amount of antibiotics-free cBD medium supplemented with 50 nM EC 23 (Table 1) to reach the desired MOI.

***Note:*** The OD factor differs per bacterial strain, and can be calculated using methods detailed by Penuelas-Urquides et al.^5^

***Optional:*** Suspensions of heat-killed bacteria at the desired MOI can be prepared using this protocol as well. In that case, include the following step after collecting bacteria in centrifuge tubes, but before centrifugation: heat-kill bacteria by incubating for 30 min at 80°C, for example in a water bath.

***Note:*** Mycobacteria infect bronchial epithelial cells with low efficiency, so a high multiplicity of infection (compared to what is used for myeloid cell infection) is required to obtain sufficient infected cells for subsequent readouts.^2^^,^^6^ We routinely use MOI 100. At MOI 10, we observed that infection percentages to be very low. At MOI 1000 we did not obtain significantly more infected cells compared to MOI 100 Unpublished data.

**CRITICAL:** The mycobacterial cultures should be visually homogeneous to achieve accurate OD_600_ measurements. Cultures with clumps cannot provide reliable estimates of the desired multiplicity of infection. See the troubleshooting section for solutions to clumping cultures. Mycobacteria should be in log phase (OD_600_ 0.2–1.0) at the time of preparing bacterial suspensions, to ensure optimal growth conditions.

25. Plate the bacterial suspensions on 7H10 agar to be able to retrospectively determine the accuracy of the prepared MOI.
  a. Make serial 1:10 dilutions in PBS. The number of dilutions depends on the chosen MOI (example provided for MOI 100 suspension in Table 2).
  b. Plate 10 μL drops of each dilution on the dried 7H10 agar plates in triplicate.
  c. Allow the drops to be absorbed by the agar, then seal the plate with tape along the edges to prevent dehydration of the agar during incubation.
  d. Incubate 7H10 plates in a dry incubator at 37°C.
  e. Check bacterial growth frequently and take plates out of the incubator when colonies are visible. The incubation time can vary between species. For example, 3–5 days for *M. smegmatis*, 10 to 15 days for *M. avium* and *M. bovis* and 14–21 days for *M. tuberculosis.*
26. Right before adding bacteria, wash the apical side of ALI-PBEC cultures to remove the mucus layer.
  a. Add 200 μL warm (37°C) PBS to each insert.
  b. Incubate inserts 10–15 min at 37°C.
  c. Carefully remove PBS with a pipette without touching the insert.
27. Add 50 μL bacterial suspension to the apical side of the inserts. Do not add bacteria to the basal compartment.

***Note:*** We previously determined that 50 μL is the optimal infection volume when using 12-wells plate inserts of 1.13 cm^2^. Using this volume, bacteria can spread evenly over the insert, and ALI-PBEC mucociliary function is not affected drastically. Uninfected control cultures should receive the same volume of antibiotics-free cBD medium supplemented with 50 nM EC 23.

28. Centrifuge the plates with infected ALI-PBEC cultures for 2 min at 300 × g to spin down the bacteria towards the cells.

***Note:*** Centrifuging directly after adding bacterial suspension to ALI-PBEC brings the bacteria in direct contact with the cells.

29. Incubate ALI-PBEC cultures at 37°C for 24 h.
30. After 24 h incubation, remove bacteria and mucus from the cultures.
  a. Add 150 μL warm (37°C) PBS per insert to the apical side of the ALI-PBEC cultures. Combined with the 50 μL infection volume, the total volume on the insert will now be 200 μL.
  b. Incubate ALI-PBEC for 15 min at 37°C to remove mucus.
  c. Remove apical PBS and basal medium from ALI-PBEC cultures with a pipette.
31. Eliminate extracellular bacteria by gentamicin treatment.
  a. To the basal compartment, add 1 mL warm (37°C) antibiotics-free cBD medium supplemented with 50 nM EC 23.
  b. Add 50 μL gentamicin (30 μg/mL in PBS) to the insert apically, incubate 15 min at 37°C to kill remaining extracellular bacteria.
  c. Remove gentamicin with a pipette and subsequently wash cultures once with 200 μL warm (37°C) PBS.
32. Incubate cultures at 37°C for at least 24 h before proceeding. Hereafter, change basal medium three times per week and each time wash the apical side with warm (37°C) PBS.

***Note:*** We recommend incubating PBEC with bacteria for a minimum of 24 h to ensure successful invasion of epithelial cells. We have successfully performed infections up to 10 days with Mtb and Mav. However, potential disruption of mucosal barrier integrity and cell death should be considered during longer infections.

**Table 1:** Example of MOI calculations

Dilution	OD_600_	Undiluted OD_600_	Average OD_600_	OD factor	Bacteria / mL	MOI 100 required bacteria / mL	Dilution factor	mL of culture	Resuspend in (mL)
1	0.600	0.600	0.660	1.3 × 10^7^	8.5 × 10^7^	2 × 10^9^	0.043	5	0.215
1:2	0.340	0.680							
1:5	0.140	0.700							
**Calculations**					= Avg. OD × OD factor	= 10^6^ cells^a^ × 100 × 20^b^	= $\frac{req.\;bact.}{bact.\;per\;ml}$		= dilution factor × mL of culture

a On average, an insert carries 10^6^ epithelial cells after two weeks of mucociliary differentiation.

b 50 μL volume is used per ALI-PBEC culture. Each mL contains 20 × 50 μL.

**Table 2:** Serial dilutions to retrospectively determine actual MOI

**Dilution**	undiluted	10^–1^	10^–2^	10^–3^	10^–4^	10^–5^	10^–6^	10^–7^
**CFU**	2 × 10^7^	2 × 10^6^	2 × 10^5^	2 × 10^4^	2000	200	20	2

### Part 4: Determination of intracellular bacterial load using flow cytometry

**Timing: 2–3 h**

When determining bacterial load in cells, it is useful to know both the percentage of cells infected and the actual number of intracellular colony forming units (CFU), representing live replicating bacteria. We therefore often combine flow cytometry and CFU assays (Part 5) to analyze infection efficiency of mycobacteria in epithelial cells. In addition, confocal microscopy (Part 6) is used to confirm intracellular presence of the bacteria. When working with Mtb, all steps should be performed at BSL-3 containment laboratories, unless specifically stated.

***Note:*** For Flow cytometry, single cell suspensions of ALI-PBEC cultures are produced and measured by flow cytometry to determine the percentage of infected cells based on bacterial fluorescence.

33. Incubate ALI-PBEC cultures with PBS to disrupt epithelial barrier resistance. PBS should be calcium-free.
  a. Remove basal cBD medium.
  b. Add 1 mL warm (37°C) PBS basally and 200 μL apically to each insert.
  c. Incubate for 30 min at 37°C.

***Optional:*** Basal medium and apical wash can be collected in tubes or plates at this point to study host secretome upon infection. Store supernatants at −20°C up to 6 months.

34. Prepare a tube with TrypLE Express, add 10 μM Y-27635, and pre-warm to 37°C.
35. Remove all PBS and incubate inserts apically with 200 μL warm TrypLE Express for 10–15 min at 37°C.

***Note:*** TrypLE Express is a gentle cell dissociation reagent that does not require inactivation, but is instead inactivated by dilution alone. Y-27635 is a Rho kinase inhibitor that prevents cells from undergoing apoptosis as a result of cell dissociation.^7^ Using these reagents ensures optimal conservation of the sample.

36. Transfer the single cell suspension from the insert to 1.5 mL tubes.
  a. For every insert, prepare a sterile 1.5 mL tube with 800 μL PBS.
  b. Using a 200 μL tip, pipet the TrypLE Express liquid up and down on the insert to break down remaining clumps of cells, then transfer to the 1.5 mL tubes.
  c. Rinse inserts one time with PBS from the prepared tubes to collect any cells remaining on the insert. The total volume of the sample will be 1 mL.
37. Transfer 100 μL of each sample in new 1.5 mL tubes for CFU plating (see step 25). Use remaining 900 μL for flow cytometry.
38. Prepare an unstained control sample.
  a. Collect 100 μL of an uninfected ALI-PBEC sample in a 1.5 mL tube.
  b. Add 800 μL PBS. Centrifuge 395 × *g* for 10 min at 18°C–21°C. Remove supernatant.
39. Add 400 μL 4% paraformaldehyde (PFA) for 30–60 min.
40. Add 1 mL PBS to wash. Centrifuge 395 × g 10 min.
41. Resuspend cells in 400 μL FACS buffer (0.1% BSA solution in PBS). If preferred, samples can also be measured directly in fixative instead of FACS buffer.

***Note:*** When using a spectral flow cytometer, such as the Cytek Aurora, the unstained sample is critical for unmixing fluorescence signals, as the cytometer determines background autofluorescence signals from this sample.

42. Prepare a Live/Dead single stain control sample.
  a. Collect two times 100 μL of an uninfected ALI-PBEC sample in separate 1.5 mL tubes.
  b. Heat-kill the cells by incubating one of the tubes for 30 min at 56°C. Alternatively, heat to 70°C for 10 min.
  c. Add the heat-killed cells to the tube of live cells to obtain a mixed sample of live and dead cells.
  d. Add 800 μL PBS.
43. Prepare a bacterial fluorescence single stain control sample.
  a. Take 200 μL from each bacterial culture in separate 1.5 mL tubes.
  b. Centrifuge 10 min at 805 × g. Remove supernatant.
44. Centrifuge all samples 10 min for 395 × g. Carefully discard supernatant.

***Note:*** ALI-PBEC cell pellets will easily detach from the tubes during removal of supernatant but will remain firmly aggregated. If pellets become dislodged, carefully pipet around them to remove supernatant.

**Figure 1:**
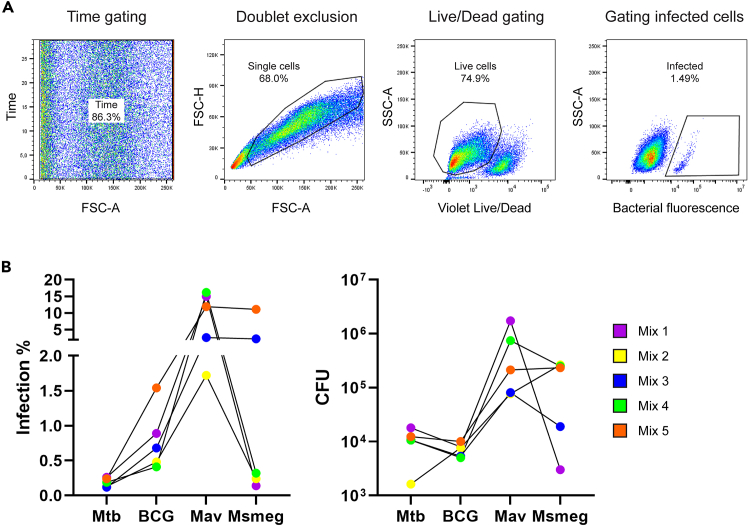
Expected results from flow cytometry and CFU experiments 48 h post infection (A) Gating strategy used to determine infection percentage. (B) Infection percentages and intracellular CFU from 5 ALI-PBEC mixed donor pools each including 4 independent donors, infected with different species of mycobacteria. Data from Barclay et al.^2^ Abbreviations: Mtb (*M. tuberculosis*), BCG (*M. bovis* Bacille Calmette-Guerin), Mav (*M. avium*), Msmeg (*M. smegmatis*).

45. Stain all samples apart from the unstained control sample and the bacterial single stains (step 14–22) with Fixable Violet Live/Dead stain.
  a. Prepare an 800 times dilution of Violet Fixable live/dead stain in PBS.
  b. Resuspend cell pellets in 100 μL of the live/dead stain.
  c. Incubate for 30 min at 18°C–21°C in the dark, then add 1 mL PBS to wash. Samples only need to be protected from light during this staining step, as they will be fixed and measured immediately afterward.
  d. Centrifuge 395 × g for 10 min, remove supernatant.
46. Add 400 μL 4% paraformaldehyde (PFA) for 30–60 min.
47. Add 1 mL PBS to wash. Centrifuge 395 × g 10 min.
48. Resuspend cells in 400 μL FACS buffer (0.1% BSA solution in PBS). If preferred, samples can also be measured in fixative instead of FACS buffer.
49. Add samples to FACS tubes with a filter cap to remove aggregates of cells.
50. Measure samples on a spectral flow cytometer such as the Cytek Aurora.
51. Obtain infection percentages using a similar gating strategy as depicted in Figure 1A. First, place a time gate to ensure no artifacts of disrupted flow are included in the data. Then, place a single cell gate to exclude aggregates of multiple cells from analysis. Next, place a gate around the live cells (unstained by the Fixable Violet Live/Dead stain), and finally gate the infected cells based on bacterial fluorescent signal.

**CRITICAL:** Because human bronchial epithelial cells have a relatively high level of autofluorescence, it is recommended to make use of a spectral flow cytometer which can compensate for autofluorescence. For this compensation, it is recommended to use cells to prepare single stains, and not beads.

***Note:*** Infection percentages vary between mycobacterial species, and different donors and donor mixes. Expected percentages are listed in Figure 1B.

### Part 5: Determination of intracellular bacterial load using colony-forming units assay

**Timing: 1 h, 3 weeks incubation**

For the CFU assay, the set-aside single cell samples collected in step 37 are prepared for cell lysis to release intracellular bacteria. Lysates are plated on agar plates to allow bacterial colony growth.

52. Treat single cells with gentamicin to kill extracellular bacteria.
  a. Add 200 μL gentamicin (30 μg/mL in PBS) to tubes containing cells.
  b. Incubate for 15 min at 37°C.
  c. Add 700 μL PBS to tubes to wash.
  d. Centrifuge 10 min at 395 × g.
  e. Carefully remove supernatant, the cell pellet is quite small.
53. Lyse cells to obtain intracellular bacteria.
  a. Resuspend pellets in 200 μL 0.05% SDS solution in Milli-Q water.
  b. Incubate for 10 min at 18°C–21°C.
  c. Add 800 μL PBS to wash.
  d. Centrifuge 10 min at 805 × g. Remove supernatant.
54. Plate lysates on agar plates.
  a. Resuspend lysates in 100 μL PBS.
  b. Make six times 1:10 serial dilutions of the lysate in PBS. The number of dilutions should be estimated based on previous experiments.
  c. Plate 10 μL of the dilutions on dried 7H10 agar plates.
  d. Allow the drops to be absorbed by the agar, seal the plate with tape along the edges.
  e. Incubate in a dry incubator at 37°C.
55. Check the plates frequently and count colonies when they become visible.

***Note:*** Mycobacteria can form rough unevenly shaped colonies that are difficult to count. Making a digital scan of the CFU plate at different time points is recommended for reference during CFU counting. Any apparatus suitable for scanning paper documents can be used for this purpose.

### Part 6: Visualization of intracellular bacteria using confocal microscopy

**Timing: 3 days**

For confocal microscopy, ALI-PBEC cultures infected with mycobacteria are fixed and stained with antibodies for analysis by confocal microscopy. By staining the epithelial cell membrane marker EpCAM, it is possible to determine whether bacteria are present intracellularly or extracellularly.

56. Fix infected inserts with 4% PFA.
  a. Wash inserts with PBS (0.5 mL apical side, 1.5 mL basal side) to remove traces of mucus and medium.
  b. Add 500 μL PFA apically and 1 mL basally.
  c. Seal the plate with tape along the edges.
  d. Incubate at least 24 h at 4°C.
57. Remove fixative.
  a. Wash inserts once with PBS, 500 μL apically and 1 mL basally.
  b. Add new PBS to each insert and well, 500 μL apically and 1 mL basally.
  c. Store cultures at 4°C in PBS and use within 6 months or proceed immediately with fluorescent staining.

***Note:*** After 24 h fixation, samples containing Mtb can be transferred to BSL-1 for further handling. If storing fixed inserts for a long time, regularly check if both sides of the insert are submerged in PBS. Do not let the cells dry out, as this will affect the quality of the staining.

***Optional:*** For intracellular staining, permeabilization of the cells is required. For this, add ice cold methanol for 10 min at 4°C (500 μL apical, 1 mL basal). This step should be performed after fixation but before blocking the inserts. Follow the required safety regulations when working with methanol.

**Figure 2:**
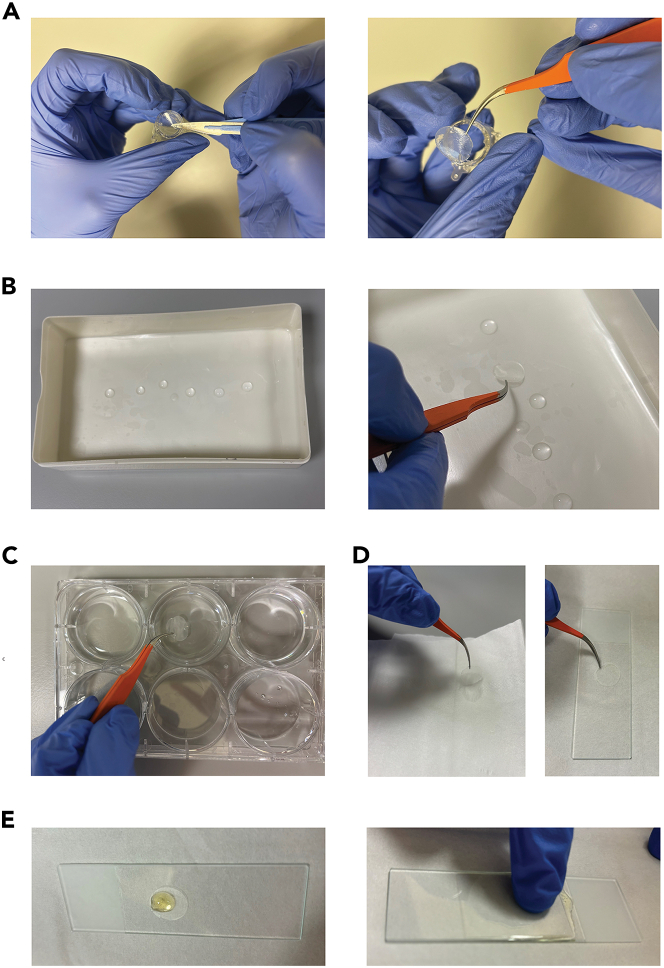
Visualization of sample processing for microscopy (A) Removal of the plastic insert by cutting the membrane containing cells. (B) Preparation of a light proof box with antibody solution droplets on parafilm, and placement of the membrane on these droplets. (C) Washing of membranes by swirling in PBS. (D) Removal of excess liquid from the membrane by blotting on tissue paper, and placement of membrane on a glass microscopy slide. (E) Addition of antifading reagent on the membrane and coverage with cover slip.

58. Block the inserts with blocking buffer.
  a. Wash inserts once with PBS (500 μL apical, 1 mL basal).
  b. Add 500 μL blocking buffer (1% BSA, 0.3% Triton-X-100 solution in PBS) apically and 1 mL basally to each insert.
  c. Incubate 10 min at 18°C–21°C or 30 min at 4°C.
59. Prepare primary antibodies.
  a. Cut a piece of parafilm and fit it inside a lightproof plastic box with a lid.
  b. Dilute EpCAM primary antibody in blocking buffer to a concentration of 10 μg/mL (20 times dilution from 200 μg/mL stock).
  c. Add 50 μL drops containing primary antibodies on the parafilm.
60. Cut out the membrane from the inserts (Figure 2A).
  a. Invert the insert.
  b. Carefully excise the membrane by slicing around the edges with a scalpel.
  c. If performing multiple stainings, cut the membrane in halves or quarters.
61. Incubate membranes with primary antibodies (Figure 2B).
  a. Using fine tweezers, place membranes on the drops containing primary antibodies with cells facing the liquid.
  b. Close the lid of the box.
  c. Incubate membranes for 1 h at 18°C–21°C or 12–16 h at 4 °C.

***Note:*** For the EpCAM antibody used in this protocol, 12-16 h incubation at 4 °C results in more specific antibody binding than incubation at 18°C–21°C.

62. Prepare secondary antibodies.
  a. Prepare a second box with parafilm as in step 59a.
  b. Dilute secondary donkey-anti-goat Alexa Fluor 405 antibody 200 times in blocking buffer to a concentration of 10 μg/mL (from 2 mg/mL stock).
  c. Add 50 μL drops on a piece of parafilm.

***Note:*** If desired, additional stains like cell viability or DNA stains can be added to step 62. Secondary antibodies and fluorescent stains can be combined in one tube. Sequential staining is not required in most cases.

**Figure 3:**
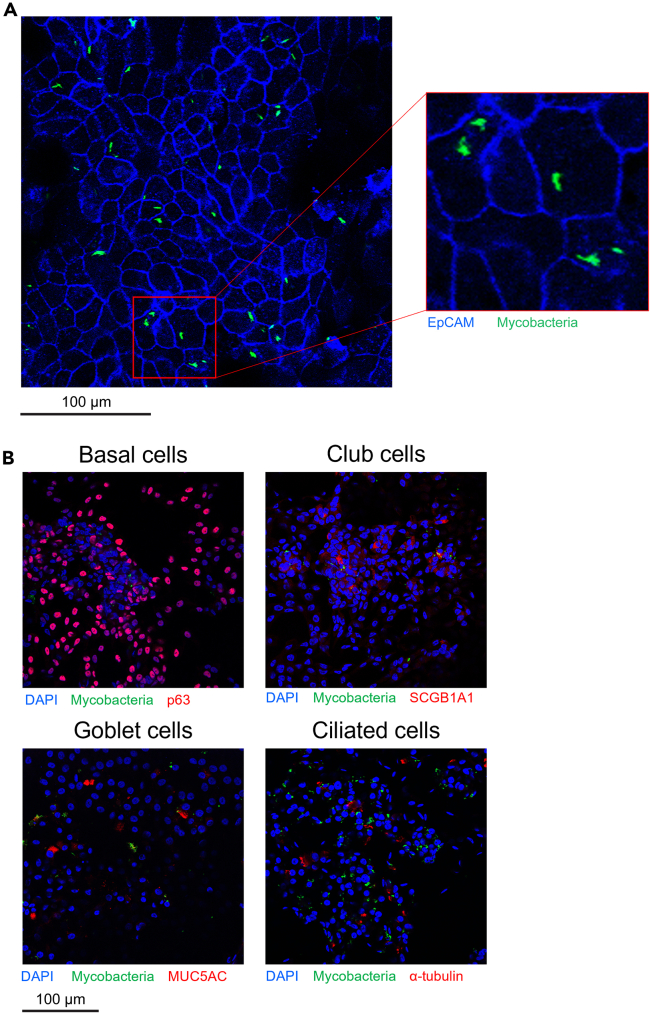
Expected results from microscopy experiments ALI-PBEC were infected for at least 24 h with mycobacteria. (A) Intracellular mycobacteria inside ALI-PBEC. Bacteria in green, cell membranes in blue (EpCAM staining). Data from experiments as published in Barclay et al, unique image from a not previously published donor.^1^^,^^2^ (B) Examples of cell type marker stains in red. Scale bars indicate 100 μm.

63. Wash excess primary antibody solution from the membranes (Figure 2C).
  a. Fill three wells of a 6-well culture plate with PBS.
  b. Carefully grab the edge of a piece of insert with tweezers.
  c. Wash the membrane pieces three times by slowly dipping them in the PBS and swirling them around.
64. Incubate membranes with secondary antibodies and stains.
  a. After washing, immediately place membrane pieces with cells facing the drops containing secondary antibodies. Immediately close the lid of the box.
  b. Incubate in the dark for 30 min at 18°C–21°C or 2 h at 4°C.
65. Wash excess secondary antibody solution from the membranes.
  a. Fill three wells of a 6-well plate with PBS and three wells with Milli-Q water.
  b. Wash membranes three times by swirling in PBS.
  c. Wash membranes three times by swirling in Milli-Q water.
66. Mount membranes on microscopy slides.
  a. Lightly dab a corner of the membrane onto tissue paper to remove excess liquid (Figure 2D).
  b. Place membrane on a glass microscopy slide with cells facing upwards (Figure 2D).
  c. Add a drop of anti-fading reagent on top of each membrane piece (Figure 2E).
  d. Cover membranes with a cover slip.
  e. Firmly press the cover slip to remove air bubbles trapped below the glass (Figure 2E).
  f. Allow anti-fading reagent to set 12–16 h at 18°C–21°C in the dark.
  g. Store microscopy slides at 18°C–21°C in a dark dry place.
67. Image membranes using a confocal microscope. Examples of staining results are shown in Figure 3A.

***Optional:*** Specific cell types can also be stained using this protocol. We have previously stained cell types using the following markers: p63 for basal cells, SCGB1A1 for club cells, Muc5ac for goblet cells and acetylated α-tubulin for ciliated cells. Examples are given in Figure 3B.

## Expected outcomes

This protocol was developed to infect human bronchial epithelial cells with *Mycobacterium* spp. Infection percentages and intracellular CFU differ per species, as some species are more efficient at infecting epithelial cells than others. Furthermore, the donor variation may also influence the efficiency of infection since some donors are more susceptible to infection while others are more resistant. On average, with the species of mycobacteria that we have tested (Mtb, BCG, Mav and Msmeg), we achieve between 0.5–2% infected cells and 10^4^–10^5^ intracellular CFU in 48 h of infection. All mycobacterial species tested were confirmed by fluorescent microscopy to infect epithelial cells intracellularly. However, this may differ for other mycobacterial species.

## Limitations

A limitation of this procedure is the low efficiency of some mycobacteria to infect epithelial cell cultures, which may hamper data collection by flow cytometry and microscopy. Furthermore, while mycobacteria are slow growing bacteria compared to other species, they do multiply during longer infection times. This should be considered particularly when comparing the infection potential of different species in epithelial cells. Finally, the composition of cell types in cultures of different donors may vary, which in turn can influence the outcome of infection.

## Troubleshooting

### Problem 1

Autofluorescence of epithelial cells interfering with flow cytometry measurements (step 50).

### Potential solution 1

Use a spectral flow cytometer that can compensate autofluorescence, for example the Cytek Aurora.

### Problem 2

Mycobacteria are forming clumps in culture (step 10–11).

### Potential solution 2

Make sure that there is sufficient Tween-80 in the 7H9 broth. Because Tween-80 is a viscous liquid, check that the correct amount is being pipetted during 7H9 medium preparation. Alternatively, split bacterial cultures to a lower OD_600_ the day before experiments, for example OD_600_ = 0.125.

### Problem 3

Lack of access to BSL-3 facilities to culture Mtb.

### Potential solution 3

Non-virulent Mtb strains like H37Ra or other related species like BCG or Msmeg can be cultured at BSL-2 facilities and can be used as model organisms.

### Problem 4

Bacteria are migrating to the basal compartment during infection (step 29–32).

### Potential solution 4

This suggests that bacteria are disrupting the epithelial cell layer. Shorten infection time or decrease the MOI.

### Problem 5

Bacteria are aggregating on the cell layer during infection (step 29–32).

### Potential solution 5

Extracellular aggregations should be removed prior to gentamicin treatment, as aggregated bacteria may not be killed efficiently by antibiotics. After the desired infection time, resuspend aggregations carefully in 500 μL PBS and remove liquid from the ALI-PBEC insert. Then continue with gentamicin treatment as described in the protocol.

### Problem 6

PBEC are not confluent on inserts after the expected incubation time (step 19).

### Potential solution 6

Incubate PBEC submerged for a few extra days until confluent, then expose to air as described.

### Problem 7

Lack of or limited access to patient materials for isolating bronchial cells.

### Potential solution 7

Commercially available primary bronchial cells can be also used.

### Problem 8

A low yield of primary bronchial cells from cryopreserved stocks (step 17).

### Potential solution 8

A smaller number of cells can be seeded per insert or inserts with a smaller diameter can be used. This way the small number of cells can be used efficiently.

## Resource availability

### Lead contact

Further information and requests for resources and reagents should be directed to and will be fulfilled by the lead contact, Simone A. Joosten (S.A.Joosten@lumc.nl).

### Technical contact

Technical questions on executing this protocol should be directed to and will be answered by the technical contacts, Amy M. Barclay (A.M.de_Waal@lumc.nl) or Kimberley V. Walburg (K.V.Walburg@lumc.nl).

### Materials availability

This study did not generate new unique reagents.

### Data and code availability

All data presented are derived from the same experiments as reported in Barclay et al.^2^ The microscopy data sets supporting the current study have not been deposited in a public repository because of their large file size, but are available from the corresponding author on request.

## Acknowledgments

This work was supported by a grant from the Leiden-Edinburgh Joint PhD program for Integrated One Health Solutions, awarded by the universities of Leiden and Edinburgh in December 2019. The graphical abstract for this publication was created with BioRender.com.

## Author contributions

A.M.B. optimized the infection and flow cytometry protocols, collected and analyzed data, and drafted the article. K.V.W. optimized the mycobacterial culture protocols and collected data for flow cytometry and colony-forming unit assays. D.K.N. optimized PBEC cell culture protocols and performed all cell culture work to create the PBEC *in vitro* models. P.S.H., T.H.M.O., A.M.v.d.D., and S.A.J. served as scientific advisors and critically reviewed the protocols, study design, and article.

## Declaration of interests

The authors declare no competing interests.
